# Exploring marriage beliefs from the perspectives of married students

**DOI:** 10.3389/fpsyg.2025.1481905

**Published:** 2025-02-20

**Authors:** Efat Sadeghian, Seyed Ali Ghasemi, Maryam Maddineshat

**Affiliations:** ^1^Chronic Diseases (Home Care) Research Center, Institute of Cancer, Hamadan University of Medical Sciences, Hamadan, Iran; ^2^Department of Nursing, School of Nursing and Midwifery, Hamadan University of Medical Sciences, Hamadan, Iran; ^3^Iranian Reality Therapy Association, Tehran, Iran; ^4^Department of Nursing, School of Malayer Medical Sciences, Hamadan University of Medical Sciences, Hamadan, Iran

**Keywords:** marriage, beliefs, background, students, marital satisfaction, spousal relationships, couple therapy, reality therapy

## Abstract

**Introduction:**

Iranian university students have a positive outlook on marriage; however, certain beliefs may impact their satisfaction with marriage and the quality of their relationships. By examining these beliefs and considering the experiences of married students, valuable information can be gathered to develop policies that protect family rights and support the younger generation.

**Aim:**

This study aimed to explore the beliefs about marriage from the perspectives of married students.

**Method:**

A qualitative study was conducted. University students were selected through purposive sampling at Hamadan University Medical Sciences,…, Iran. Data was collected from October 2022 to January 2023. Semi-structured interviews were held with 24 married students, and the analysis was conducted using conventional content analysis.

**Results:**

In our research, we thoroughly analyzed 51 beliefs about marriage held by students, categorizing them into eight subcategories, including “empathy and simplifying life,” “keeping affection alive,” “effective and transparent communication,” and others. These beliefs were further condensed into three overarching categories: “intimacy and companionship,” “establishing a secure environment,” and “equity and upholding preexisting convictions.”

**Conclusion:**

The research explored the intricate interplay of beliefs shaping university students' beliefs toward marriage. Beliefs surrounding empathy, simplifying life, sustaining affection, and effective and transparent communication contribute to marriage intimacy and compassion. Notably, beliefs concerning security, commitment, and conflict resolution strategies were identified as pivotal for creating a safe marital atmosphere. Furthermore, the presence of beliefs supporting women's autonomy was deemed crucial for fostering equality within marital partnerships, while beliefs endorsing male dominance, religious biases, sexism, and cultural bigotries were found to impact spousal relationships potentially detrimentally.

## 1 Introduction

Marriage is an intimate and profound union between two individuals who choose to spend the majority of their lives together. It is one of the most complex and meaningful human relationships that requires both partners' deep understanding and commitment (Girgis et al., 2020). Many couples often start their marriage with strong love but may not realize that the intense passion they initially feel for each other may fade over time. Ellis stated that (Ellis et al., 1989) the decline of romantic love is a common occurrence that newlyweds rarely anticipate. In the context of marriage, individuals often hold distinct beliefs and assumptions regarding the dynamics of their relationship, the responsibilities of their partner, and the desired outcomes of the relationship. These beliefs also encompass interpretations of different scenarios, challenges that may arise within the relationship, and expectations for the behavior required to maintain a successful partnership (Sullivan and Schwebel, 1995). Marriage beliefs play a crucial role in shaping the meaning individuals associate with marriage, ultimately impacting their behavior as a couple. These beliefs can influence how a couple manages their relationship (Mohammadi and Soleymani, 2017).

In married life, rational beliefs refer to an individual's understanding and beliefs about their relationship. These beliefs can significantly impact their decisions, actions, and overall satisfaction with their marriage. Research has shown that holding irrational beliefs can lead to marital dissatisfaction and conflict behavior (Adibkia et al., 2022). Ellis et al. (1989) was a pioneer in highlighting the significance of irrational beliefs in the breakdown of marital relationships. He believes that irrational beliefs held by spouses, not only about themselves and others but also about marriage, are the root cause of dysfunctional marriages. Irrational beliefs are characterized by being exaggerated, unrealistic, rigid, and resistant to change. They are not grounded in personal experiences and are often viewed as inconsistent, unhealthy, and dysfunctional. These beliefs are impractical, and synonyms for irrational beliefs include inconsistent, unhealthy, and dysfunctional (David et al., 2009).

Ellis et al. (1989) have identified irrational beliefs in marital relationships, including unrealistic expectations of spouses, feeling worthless without their partner's affection, and exaggerating the situations they face. Lazarus (1985) identified 24 irrational beliefs about marriage in her book “Marriage Myths” that can diminish marital satisfaction. Many individuals with irrational beliefs about marriage turn to psychological centers for advice and guidance. Among those seeking help, students seem to be a prominent demographic (Yaman and Yavuz, 2020). College students' beliefs about marriage can impact their relationships. Those with positive beliefs toward marriage tend to have higher levels of relationship self-efficacy. Factors such as age, gender, and race also play a role in influencing students' attitudes toward marriage (Shurts and Myers, 2012). Studies have been conducted to measure college students' beliefs in myths about marriage and family relations, which suggests that these beliefs can impact their perceptions and expectations within relationships. It is crucial to consider the influence of these beliefs on the dynamics of relationships and decision-making among college students (Musick et al., 2012; Larson, 1988).

During the transition to adulthood and marriage preparation, a significant aspect of romantic relationships is formed during the college student period. This time is crucial to determine the quality of relationships with a partner and to make an informed decision when choosing a spouse in the following years (Nadzirah et al., 2018). For three reasons, Greenberg and Nay (1982) highlighted the significance of acquiring information about college students' beliefs regarding marriage. Firstly, the current beliefs of college students about marriage are not known. Secondly, it is important to understand their beliefs regarding the age at which they plan to get married, their expectations from marriage, their behavior in marriage, and ultimately their satisfaction with married life. Lastly, identifying their beliefs about married life helps determine family life education content. Cultural and societal factors such as peers, religious institutions, media, education, and cultural trends significantly shape college students' beliefs about marriage, which can vary based on their individual backgrounds and experiences (Xie and Hong, 2022).

Marriage in Iran is deeply intertwined with Islamic beliefs. It is considered a sacred institution that completes one's faith. The literature indicates that many Iranians view marriage as essential for spiritual growth and societal acceptance. Marriage is often viewed through a traditional lens, emphasizing stability, social responsibility, and religious significance (Rahbari, 2019; Keshavarz et al., 2018). This reflects a broader cultural narrative in which marriage is seen as a cornerstone of both individual identity and social structure (Soulsby and Bennett, 2017). Several similarities and differences emerge when comparing Iranian attitudes toward marriage with those from other countries. In many Islamic societies, such as those in the Middle East and North Africa, marriage is similarly viewed as a religious duty. Studies across these regions have documented pressures on individuals to marry early and within specific cultural norms (Hajiheydari and Pourshahriari, 2024; Al Akash and Chalmiers, 2021; UNICEF MENA, 2017).

In contrast, Western attitudes toward marriage prioritize romantic love and personal choice, viewing marriage as a partnership based on mutual respect and equality. This perspective diverges significantly from collectivist cultures, where family approval and social obligations play a more substantial role in partner selection (Karney, 2021). Shown in Research from Western countries is a trend toward later marriages and cohabitation before marriage as socially acceptable practices. This divergence highlighted how cultural contexts shape beliefs about marriage; while Western societies may prioritize personal fulfillment and egalitarian relationships, Iranian society still grapples with traditional expectations (Sassler and Lichter, 2020; Aghajanian et al., 2018).

Traditionally, marriages in Iran are arranged by families, focusing on social status and familial connections. However, contemporary views emphasize personal choice and compatibility in partner selection, with younger generations prioritizing emotional connections and shared values over familial expectations. This shift is particularly evident among youth with increased access to education and global cultural influences (Clyde et al., 2020). Research indicates that beliefs about marriage among Iranian youth vary based on upbringing and societal expectations, making it crucial to understand these beliefs for guiding young people through marriage and family formation amid changing norms (Khalili et al., 2022; Fallahchai et al., 2021). Additionally, Iran is experiencing a demographic shift toward an aging population due to declining marriage and fertility rates. Approximately 11.5% of the population is currently aged 60 or older, projected to rise to 32% by 2050 (Pezhhan and Ambika, 2023). This transition requires comprehensive strategies to address the needs of the elderly while promoting marriage and family formation. Despite government efforts to encourage marriage through various policies, these initiatives have seen limited success in reversing the declining trend (Gietel-Basten et al., 2024).

There are qualitative studies from Iran investigating the attitudes of unmarried young people, married students, and couples regarding marriage, primarily focusing on factors influencing these attitudes (Keshavarz et al., 2018; Mehr R. K, 2016; Shomoossi et al., 2022). In contrast, our study delves into the interplay of tradition, modernity, and individual choice in shaping marriage beliefs in Iran. While many young people view marriage as a religious necessity, there is an increasing acceptance of personal choice in marital decisions. By examining university students' beliefs, we aim to understand how personal aspirations, societal expectations, and practical challenges shape their perspectives on marriage. Although many students hold a positive view of marriage (Keshavarz et al., 2018), certain beliefs can impact marital satisfaction and relationship quality. Additionally, identifying beliefs about marriage and sharing married students' experiences can inform laws related to family rights and support for the youth. Thus, this study focuses on exploring marriage beliefs from the perspective of married students.

## 2 Methods

### 2.1 Study design

Qualitative research delves into participants' thoughts, emotions, and experiences to gain insights into human behavior and social contexts, often revealing motivations overlooked by quantitative methods (Pyo et al., 2023). We employed a qualitative approach using convection content analysis, which systematically interprets textual data through coding and theme identification. Marriage is a social phenomenon, so this method aligns with social science principles (Bengtsson, 2016; Šuvaković, 2021). We adhered to the COREQ checklist (Supplementary Table S1) in reporting our findings (Tong et al., 2007).

### 2.2 Study context

The study was conducted at Hamadan University of Medical Sciences (UMSHA), which is an ideal location due to its unique cultural context, diverse student body, and strong history of related research. This environment is particularly suitable for examining married students' beliefs about marriage. UMSHA offers various faculties and currently enrolls 6,900 students across over 70 undergraduate and graduate programs in western Iran (Hamadan University of Medical Sciences, 2023).

### 2.3 Participants and sampling

As a qualitative study, participants were recruited through a maximum variety of purposive sampling. This method seeks individuals with significant experiences related to the phenomenon who are eager to share their insights (Holloway and Galvin, 2024). Participants in this study were university students who volunteered and met the following criteria: (1) Male and female students interested in participating. (2) Must be married. (3) Must be currently studying. (4) Can be either undergraduate or postgraduate students. A total of 24 married university students (6 undergraduate and 18 postgraduate) from various faculties were selected through purposive sampling.

### 2.4 Data collection

Married university students were interviewed in a comfortable setting in faculties. The main researcher (M.M.) held one-on-one meetings in a quiet room with no one else present. The research inquiry was developed based on existing literature (David et al., 2009; Lazarus, 1985; Larson, 1988), practical knowledge, and advice from academic and professional experts. We began the interviews with a general question: “What do you expect from your spouse?” Then we asked, “Do you think your expectations were reasonable?” Through active listening and a supportive atmosphere, the interviewer (M.M.) facilitated a safe space for the participants to express their narratives and viewpoints candidly. Two individuals' insights shared in pilot interviews who were not included in the main study, along with Lazarus's framework on marital myths (Lazarus, 1985), informed the direction of the subsequent interview questions (see Table 1).

**Table 1:** Participants' interview guide about beliefs on marriage.

**Where do your beliefs come from?**
What impact do your beliefs have on marital relations?
How do you respond when your beliefs are not fulfilled?
What has led you to change your beliefs?
What measures do you take to adapt your beliefs?

During the interviews, the interviewer asked probing questions such as “Tell me more” and “Give me an example” to gather more information. Each interview lasted between 40 and 60 min, with an average duration of 50 min. We did not take any field notes during interviews because we did not use observation as our data collection method. Instead, we used different communication techniques for the interactive interviews and wrote memos and keywords during these sessions. We conducted 24 in-depth interviews with married students and reached data saturation after 21, with the last three interviews yielding no new insights. None of the participants were refused or dropped from the study. The Researchers collected data through audio recordings. Data was collected from October 2022 to January 2023.

### 2.5 Data analysis

The interviews were transcribed verbatim from Persian to English by MM and ES, and the data was transferred to MAXQDA 18 for better data management. Personal information like names and addresses was removed during transcription. To ensure accuracy, the transcription was carefully reviewed by the two research assistants and the facilitator multiple times. We employed qualitative conventional content analysis following the method outlined by Graneheim and Lundman (2004). Before coding, we carefully read all the transcripts several times to understand the issues raised. We labeled each meaningful part of the text with a code. MM and SG then assigned codes to the condensed meaningful parts to more abstractly represent the participants' words. Finally, we grouped similar codes into subcategories and categories using a process of constant comparison, reflection, and interpretation. MM discussed all subcategories and categories with ES. In case of persistent disagreement, the judgment of a third researcher (SG) was decisive.

### 2.6 Ensuring rigor

To ensure the reliability of the research findings, we continued sampling until data saturation and meticulously chose relevant units. To enhance credibility, the interviewer conducted face-to-face meetings with all participants to summarize their interviews and interpret their statements. They were invited to clarify meanings, correct inaccuracies, and offer additional insights on topics discussed in the initial interview. Feedback from this member-checking process was included in the final report. We also identified key findings from our analysis and created summaries of these categories. Subsequently, we contacted participants to encourage them to review and provide feedback on the accuracy and relevance of the key categories to their experiences. To strengthen confirmability, a comprehensive outline of the research methodology was shared with all co-authors for their approval. In this study, confirmability was obtained by avoiding any bias and agreeing on codes and themes for all research team members. Dependability was ensured by describing the analysis process. Additionally, the transferability of the findings was demonstrated as the results were logical for three married university students who did not participate in this study.

### 2.7 Ethical considerations

The study details were explained to all participants. Taking part in the study was voluntary, and students could opt to leave the study without facing any consequences. The participants' identities were kept private, and given a special code to ensure their information remained confidential. Only the researchers were allowed to access the research data. The study was conducted in line with the Declaration of Helsinki and was approved by the Ethical Committee and Research Council of Technology of Hamadan University of Medical Sciences (IR.UMSHA.REC.1401.488).

## 3 Findings

### 3.1 Demographic characteristics

Out of 24 participants, 50% were male and 50% were female. These participants were selected from different schools and had varying education degrees. Table 2 displays the demographic characteristics of the participants.

**Table 2:** Socio-demographic characteristic of participants (*N* = 24).

**School of advanced technology in medicine**	**School of paramedical**	**School of health**	**School of rehabilitation**	**School of pharmacy**	**School of dentistry**	**School of medical**	**School of nursing and midwifery**	
2	3	2	2	2	2	3	8	Number of participants
								Age categories (years)
1	1	1	2			3	6	20–30
1	2	1	1	2	2		1	31–40
							1	41–50
**Gender**								
1	2	1	1	1	1	2	3	Male
1	1	1	1	1	1	1	5	Female
**Education**								
1	1	1	1				2	Bachelor's degrees
	2	1	1				3	Master's degree
				2	2	2		MD degree
1							2	PhD degree
**During married (years)**								
	1						2	>1
1		1	1			2	2	1–5
1	1	1	1	1	1		3	6–10
	1			1	1	1		11–15
							1	16–20

### 3.2 Classification of beliefs

In our investigation, we identified and clustered 1,125 open codes representing 55 beliefs on marriage among university students into eight subgroups. This examination classified the beliefs about marriage according to the principles of Albert Ellis and Rational Emotive Behavior Therapy (REBT), linking these beliefs to core notions of “should,” “must,” “have to,” or “need to” (David et al., 2009).

Table 3 shows subcategories including “Empathy and simplifying life, “sustaining affection,” “effective and transparent communication,” “feelings of safety and commitment,” “methods for resolving marital issues,” “embracing autonomy for women,” “male dominance,” and “religion, sexism, and culture bigotries”. These beliefs were further condensed into three overarching categories: “intimacy and companionship,” “creating a secure marital atmosphere,” likewise, “equity and upholding preexisting convictions”.

**Table 3:** Illustration of subcategories and categories regarding diverse marital belief university students.

**Subcategory 1**	**Subcategory 2**	**Category**
Couples must be partners and work together.	Empathy and simplifying life	
Couples should share the responsibilities of daily life.		
Couples should communicate and make decisions together.		
Couples should make time to spend together.		
Couples should share living expenses.		
Couples should remain close to each other.		
Couples should avoid being excessively strict.		
Couples need to deal with each other conditions.		
Couples should be willing to spend money without being stingy.		
Couples should strive to create peace in their lives.		
Couples should show love and attention to each other.	keeping affection alive	
Couples should often communicate their love for each other using words.		
Love alone is sufficient to begin a marriage.		Intimacy and companionship
Each couple's lack of affection must be compensated by the other.		
To win your partner's attention and affection, should behave sulkily.		
Couples should be honest with each other.	Effective and transparent communication	
Couples should not refuse to talk.		
Couples should not hold grudges.		
Couples should talk to each other calmly.		
In discussions, couples should show respect for each other.		
Couples should communicate their expectations and requirements before getting married.		
Couples in a relationship should communicate clearly with each other.		
The bride should have a close relationship with her mother-in-law and her husband's family.		
After getting married, the couple should continue to respect their parents.		
Couples should not betray each other.	Feelings of safety and commitment	
Couples should be loyal to each other.		
Couples need to be trustworthy.		
Couples should not try to control each other.		Creating a secure marital atmosphere
Couples should read books to solve their problems.		
Couples should communicate to solve their problems.		
Couples should seek counseling to solve their problems.	Methods for resolving marital issues	
Couples should not bring their marital issues into their family relationships.		
A woman should be patient with the challenges of married life.		
In an argument, one partner should stay quiet in front of the other partner.		
A woman should not only be a homemaker.	Embracing autonomy for women	
A woman should be educated.		
A woman should not rely on her husband for financial support.		
A woman should be present in the community.		
A woman must protect her rights.		
If a woman is in an unhappy marriage, she should think about getting a divorce.		
In married life, the husband's role should be respected.	Male dominance	
In married life, the husband's respect must be maintained.		Equity and upholding preexisting convictions
In marriage, the husband's dignity should be respected.		
In married life, the husband's pride should be preserved.		
Couples should strictly adhere to their pre-marriage beliefs after getting married.	Bigotries of religion, sexism, and culture	
Men are superior to women.		
In a marriage, the woman should be younger than the man.		
Pursuing romantic relationships with the opposite sex should be avoided before or after getting married.		
Divorce should not occur solely due to sexual dissatisfaction.		
In marriage, men are responsible for living expenses.		
The husband's relatives are not good people.		
The daughter-in-law is not a good person.		

### 3.3 Intimacy and companionship

#### 3.3.1 Empathy and simplifying life

This subcategory includes a collection of beliefs shared by couples centered on two main ideas. The first concept is empathy, where couples value togetherness, engaging in activities, making joint decisions, prioritizing quality time together, and not solely focusing on work. Additionally, they believe in sharing living expenses, fostering emotional closeness, mutual understanding, and accepting each other's circumstances.

In this regard, one of the participants said:

“*Husband and wife complement each other. While both have privacy and personal goals, they must share common ground in some aspects of life. This implies that they should be on the same journey and have aligned goals in life...” (p2)*.

The concept of “simplifying life” is deeply rooted in couples' beliefs. By simplifying their lives, couples purposefully deliberately eliminate surplus and avoidable stress, allowing them to focus on their priorities, values, and what truly matters to them. Couples understand the importance of flexibility, acceptance of their partner's circumstances, and the effort to minimize conflicts to achieve peace. While some argue that couples prioritize money over their comfort, many believe in the value of creating a peaceful and harmonious life together. They can spend money but opt not to. One participant said:

“*She and her husband never argue about “my money” vs. “your money.” She explained that she has seen her family members use their own money to buy things for the house but then take money from their spouses…” (p3)*.

#### 3.3.2 Keeping affection alive

Preserving marital affection necessitates the enduring presence of love, which is essential for marital satisfaction, providing joy, support, and a deep sense of connection with one's partner. Couples rely on receiving love and support from each other throughout their relationship. Some people expect their partners to love them as much as they love their children, stressing the importance of mutual affection. Certain couples believe that verbal declarations of love become redundant after marriage, arguing that actions should suffice to convey affection without verbal expressions. Verbal and physical affection, including affectionate touch, are crucial in maintaining relationships and enhancing positive experiences. Therefore, it is vital to continue expressing love verbally and through actions to nurture and sustain marital bonds. As illustrated by a participant's regret over underestimating the importance of love post-nuptials, highlighting the significance of affectionate communication in marital dynamics, One participant explained, “*...After getting married, I discovered that my wife has an inflexible temper and does not value love as much. I wish I had been more careful in choosing a partner...” (p4)*.

Some couples experienced a lack of parental affection during childhood, leading them to seek compensation for this deficit from their spouse in adulthood. Failure to address this emotional gap within the marriage can result in conflicts between partners. This compensatory behavior was exemplified by a participant recounting how their husband's childhood devoid of maternal love hindered his ability to form connections in adulthood, turning to the spouse for the affection he lacked. Additionally, couples may avoid meaningful conversations as a misguided strategy to elicit love and attention from their partner, viewing it as a protective measure despite recognizing its ineffectiveness.

One participant explained,

“*…I get angry easily and don't talk to my wife when I feel upset. I read somewhere that kids sulk for love and attention from their moms. This might have influenced me in my childhood…” (p11)*.

#### 3.3.3 Effective and transparent communication

This belief involves couples valuing honesty, communicating clearly, speaking calmly, and staying close to the husband's family. It also means not holding onto grudges, clearly stating expectations and needs, and continuing to honor their parents. Being honest in a romantic relationship is about being real and sincere with your partner. It involves sharing thoughts and feelings openly without hiding or manipulating words. One participant mentioned,

“*…My husband is always truthful; we share everything openly...” (p12)*.

Couples should communicate clearly to ensure their partner understands them. When discussing problems, it's important to talk calmly and show respect. Sometimes, men see the relationship between a mother-in-law and daughter-in-law negatively. Some brides struggle to establish a deep emotional connection and avoid forming close bonds. The strength of this connection can significantly impact the overall quality of the marriage. To cultivate a lasting and fulfilling marriage, husbands believe that brides should strive to develop a stronger and more intimate relationship with their husbands' families. One participant mentioned,

“*…I explained to my wife that when we got married, our lives merged. Your parents' pain is my pain, and I worked to change her views and interactions with my family…” (p9)*.

Also, after getting married, some young couples believe it is crucial to continue showing respect toward their parents. However, it is equally important for the couple to assert their independence and make their own decisions without being overly influenced by their parents. While parents may offer advice based on their own experiences, the couple should navigate this without allowing it to significantly impact their own lives. One participant shared,

“…*I used to think that my parents shouldn't interfere. However, now we have also set boundaries regarding the opinions of my husband's parents. While we sometimes seek their advice, we express gratitude and let them know that we will consider their input.…” (p 8)*.

### 3.4 Creating a secure marital atmosphere

#### 3.4.1 Feelings of safety and commitment

This subcategory encompasses beliefs centered on reliability, such as maintaining trust, being dependable, and avoiding attempts to exert control over others. Security and loyalty are crucial for most couples. Loyalty is demonstrated by remaining dedicated to one another, maintaining trust, and providing support through ups and downs. Relationship security means feeling safe and supported by one's partner. This sense of security helps couples tackle life's challenges together, with a solid foundation that can handle any obstacles. One participant emphasized the importance of loyalty in relationships, stating:

“…* Betrayal is a non-negotiable boundary for me. While I may be willing to forgive my partner for breaking my trust once, I will end the relationship and prioritize my well-being if it occurs again…” (p12)*.

In the beliefs of couples, a trustworthy partner is someone dependable. When a partner shows trustworthy traits, it strengthens the relationship by reassuring the other person that they can rely on them and don't need to be controlling. Another participant shared:

“…* I believe in allowing my spouse the freedom to make their own choices, as I value independence and dislike the idea of controlling others. Attempting to control someone only shows a lack of trust in them. I have chosen my spouse because I trust her and find her reliable…” (p6)*.

#### 3.4.2 Methods for resolving marital issues

Couples often face challenges after getting married. Sometimes, one partner may show behaviors that can harm the relationship. To keep a happy marriage, couples believe they should read books, conversate, seek counseling, avoid involving their families in their issues, and practice patience and tolerance. Not all couples do all these strategies together. Each couple chooses one based on their beliefs and experiences with these strategies. One participant said:

“*… Arguments can happen between couples in any family due to ignorance or differences. I believe every couple should read a book on marriage to avoid trouble...” (P5)*.

Some couples have found that communication is key in resolving their issues, and they consciously try to keep their families out of disagreements. They believe that involving relatives in arguments only complicates matters further. Some women think that if they are patient with their husbands' bad behavior, they can help them change and keep their relationship strong. In this regard, a participant said:

“*… My husband used to think I was solely responsible for chores. I put up with his aggressive behavior, thinking some tension between us was normal. Now, he helps with the housework and understands it's not just my job…” (p14)*.

Couples may stay silent during arguments to resolve the issue, but one partner may perceive it as avoiding the discussion and not addressing the main problem.

### 3.5 Equity and upholding preexisting convictions

#### 3.5.1 Embracing autonomy for women

Some participants believe that women should take care of household chores, cook, clean, raise children, and ensure a warm, clean, and welcoming home for everyone. However, most female participants in the study rejected this belief and supported women's independence from their husbands. This view is shared by most female participants and even some men. At the start of marriage, many women express to their husbands their desire to further their education and not only focus on household chores. One participant mentioned:

“*Ever since I got married, I have emphasized the importance of education to my husband. I expressed my desire to continue my studies, and he supported my decision…” (p18)*.

In this study, women are breaking free from traditional gender roles where they are confined to household chores and financial dependence on their husbands. They are advocating for their rights and pushing back against outdated societal norms. These women strive to participate in society and pursue their own careers actively. One participant mentioned*:*

…*I enjoy working, being around people, being independent, and being self-sufficient…” (p20)*.

#### 3.5.2 Male dominance

Some male couples are addressing the challenges of feeling distant when their partners fail to recognize men's authority in the marriage. This can result in reduced communication and time spent together. In traditional Iranian culture, the father or husband typically assumes a dominant role in the family. Although this belief is old, it persists in some Iranian families. Fathers pass on the concept of men's dominance to their sons, who then carry it into their marriages. Men highly value being respected and appreciated for their role in the family after marriage. They expect their spouses to acknowledge their significance in the family and value their presence at home. Someone expressed their feelings by saying:

…* Men want their wives to be proud of them. When my wife shows independence, it can make me feel like she doesn't rely on me and can handle everything independently with her job and income. Discussing this with my wife can be difficult for me at times…” (p17)*.

#### 3.5.3 Bigotries of religion, sexism, and culture

This subcategory observed profound prejudices rooted in religious, gender-based, or cultural frameworks. Bigotry entails the possession and vocalization of intense, irrational convictions. Following marriage, certain couples acknowledge the irrationality of specific beliefs they hold. For instance, some couples believe they should strictly adhere to their pre-marriage beliefs. During the early stages of marriage, some couples look to the beliefs and traditions of their parents or other family members as a model for their own married life. They strive to follow these beliefs and incorporate them into their marriage. One participant in the group said,

“*…I remember my mother's advice to sweep the house and clean household items daily. I applied this diligently in my married life…” (p16)*.

However, as time passes, they realize these strict beliefs only cause stress. Gradually, they modify their beliefs over time. The belief of male superiority, indicating the significance of being male, is evident in some participants who exhibit a sense of gender superiority, perceiving men as stronger and superior to women, prioritizing themselves over women. This sense of superiority influences behavior in marital conflicts, leading men to refrain from apologizing to women even when at fault, as they uphold their statements to preserve their masculine pride. One respondent said:

“*…I often replicated the patriarchal ethos we learned in our youth from our ancestors and older siblings. While this behavior was helpful in some situations, I now realize it was not always appropriate. For instance, I regret not showing simple affection, like holding my spouse's hand during our outings…” (p1)*.

Another belief among some participants is that a woman should be younger and subservient to a man so that he can be taken seriously. This sexist belief suggests that a woman's worth is tied to her youth and deference to male authority. One participant shared:

“*…At first, the age gap between my spouse and me wasn't a big issue. But as our relationship progressed, it became more significant. My wife saw herself as independent and didn't always agree with what I wanted…” (p19)*.

Some individuals adhere to the belief that engaging in romantic relationships with the opposite gender before or after marriage is morally incorrect, primarily driven by religious convictions. This perspective deems any form of romantic association outside the bounds of marriage as strictly prohibited and morally wrong, contradicting religious doctrines. The constraints imposed by religious and cultural norms can hinder individuals from fully getting to know their potential spouse before marriage, limiting premarital interactions and assessing compatibility. Consequently, these restrictions may impede the development of a profound understanding between partners before entering into matrimony, potentially impacting marital satisfaction. Couples adhering to stringent beliefs regarding relationships may experience heightened conflicts and stress, ultimately leading to decreased satisfaction levels, which can persist post-marriage and exacerbate feelings of discontent. This dissatisfaction may be further exacerbated by the inability to seek external emotional support due to societal taboos, intensifying marital discord. One participant said:

“*…My religious beliefs have made it hard for me to form close emotional connections with others besides my wife. I worried that developing such bonds would affect my feelings for my partner. Now, I regret not pursuing these connections…” (p24)*.

Another cultural belief that persists is that marital dyads should remain united even if they lack fulfillment in their conjugal relationship because the dissolution of marriage is considered a social taboo, particularly for the female gender, as it is perceived as a mark of disgrace within the cultural context. The participant expressed concern about the potential impact of divorce on their family's social standing, stating,

“*…Another reason why I don't want to get a divorce is our family's reputation, as our living environment is quite small. Very soon, everyone will know I divorced my wife…” (p23)*.

There is a common belief among participants, especially women, that men are expected to be the primary financial providers for their families. This expectation places the responsibility for covering most household expenses on men. This traditional gender expectation has long mandated men to shoulder the financial burden of supporting their families, while women are traditionally linked with domestic duties. Despite these entrenched beliefs, some women now view their earnings as personal resources, expecting reimbursement from their husbands if they contribute financially to the household. One male participant mentioned that his wife sometimes accuses him during conflicts, saying,

“*I bought that item with my earnings; why didn't you pay for it?” (p21)*.

Some individuals in Iran perceive that upon marriage, the mother-in-law or sister-in-law might intrude in the bride's personal affairs, leading to emotional turmoil. This intrusion can result in significant distress for the bride, impacting her well-being and marital harmony. The interference from extended family members in a woman's married life may cause the husband's aggression, affecting his mental and emotional health. As a result, some Iranian females view the husband's relatives as potential sources of disruption to the bride's domestic life. One participant expressed:

“*... my spouse held negative opinions about my family, particularly my mother and sister, perceiving them as bad individuals...” (p22)*.

Also, there is a prevalent belief in some Iranian families that the daughter-in-law is inherently negative, leading to potential mistreatment post-marriage. These families often fail to recognize the daughter-in-law as an equal family member and instead see her as a threat to their son's loyalty and affection. One participant recounted her experiences:

“*…It took considerable effort for me to convince my husband to alter his mother's behavior and reduce her involvement in my life. Despite this, my mother-in-law's beliefs remain unchanged, and it is only natural that she still does not hold me in high regard…” (p7)*.

## 4 Discussion

Our study explored marriage beliefs from the perspectives of married students. Three categories derived from our findings include intimacy and companionship (empathy and simplifying life, keeping affection alive, effective and transparent communication),” “Creating a secure marital atmosphere (feelings of safety and commitment, methods for resolving marital issues),” and “equity and upholding preexisting convictions (embracing autonomy for women,” “male dominance,” and “bigotries of religion, sexism, and culture).” We lacked sufficient studies on student marriage for comparison, so we compared our results with those of similar studies. We have presented our findings based on the categories separately.

### 4.1 Intimacy and companionship

Our study identified intimacy and companionship emerged from the marriage beliefs of students. In line with our study, recent studies affirm that intimacy and companionship are fundamental components of marital relationships, nurtured through empathy, affection, effective communication, and shared beliefs. These elements enhance emotional connections and contribute to overall marital satisfaction and stability (Tavaloli et al., 2022; Kamali et al., 2020).

Our study found that married university students cultivate intimacy and companionship in their relationships through beliefs in empathetic connections, simplifying life, nurturing affection, and maintaining transparent communication. Additionally, another study highlighted that Iranian couples perceive intimacy as a dynamic process characterized by self-disclosure, trust, and emotional closeness. Spending time together is crucial for fostering companionship and enhancing marital intimacy, while mutual engagement in activities strengthens relational bonds and shared beliefs about intimacy (Shomoossi et al., 2022). However, another study explored the connections between spiritual intimacy, marital intimacy, and overall well-being. It found that spiritual intimacy positively correlates with marital satisfaction through emotional intimacy, indicating that shared spiritual beliefs can enhance marital relational quality (Holland et al., 2016). In line with our findings, these studies collectively reinforce that in married life, beliefs encompass couples' understanding and expectations about their relationship. Two critical aspects of these beliefs are intimacy and companionship, which significantly influence the quality and stability of a marriage.

In this context, while there is a significant amount of research on beliefs surrounding the process of marriage, there has been relatively little focus on individuals' perceptions of the permanence of marriage and marital relationships. A research has shown that individuals who prioritize stability in their marriages tend to experience higher levels of marital satisfaction, which underscores the importance of intimacy and companionship in marital relationships (Karimi et al., 2019). One possible reason the category “intimacy and companionship” emerged from our participants' dialogues is the close connection between marriage and Islamic beliefs in Iran. Research indicates that traditional values, particularly among religious couples, significantly contribute to marital stability. Higher levels of dyadic cohesion and satisfaction are linked to stronger beliefs, which, in turn, enhance intimacy and companionship, especially among couples with religious backgrounds (Cabrera-García et al., 2019). Couples who share important values, especially religious beliefs, tend to experience higher marital satisfaction. A study indicates that agreement on core values leads to less conflict and a more enjoyable marriage (Parry, 2016).

### 4.2 Creating a secure marital atmosphere

Our study identified the category “creating a safe atmosphere,” which emerged from students' marriage beliefs. This category includes three subcategories: sense of safety, commitment, and conflict resolution strategies. Creating a safe environment hinges on safety, commitment, and conflict resolution, grounded in shared participant beliefs. Willoughby et al. (2017) identified six key dimensions of the marital paradigm, with Marital Centrality being crucial. Research on how individuals perceive the importance of after-marriage is limited. However, some scholars suggest that beliefs about marital centrality are significant. Many view marriage as a demanding commitment, often likening it to a “full-time job,” which highlights the personal resources needed. Thus, understanding marital centrality involves recognizing the effort individuals allocate to their marriages. Based on the Marital Centrality paradigm, our participants utilize a sense of safety, commitment, and conflict resolution strategies to help create a safe environment.

Beliefs shared between couples, particularly regarding creating a safe space, are fundamental for couples to express their thoughts and feelings openly. Research indicates that when partners feel secure, they can engage in more meaningful interactions, leading to personal growth and a stronger relationship foundation. A safe atmosphere reduces the need for hypervigilance, allowing individuals to focus on nurturing their relationships and pursuing personal goals. Believing in a safe atmosphere greatly influences couples' interactions. When safety is lacking, it can lead to negative emotions and the deterioration of the relationship (Kus Ambrož et al., 2021).

Based on our research results, the development of feeling safe and committed in relationships among Iranian couples is shaped by their belief system, which emphasizes loyalty, avoiding control, avoiding betrayal, and building trust. Iranian married couples typically prioritize loyalty and faithfulness in their relationships. Loyalty, trust, and belief in God are deemed crucial for a successful marriage in Iran (Tavakol et al., 2016). While loyalty holds significant importance in Iranian marriages, its implementation can differ based on location, educational background, and unique family dynamics. Loyalty and trust serve as essential building blocks for thriving marital relationships in Iran, despite variations in how they are demonstrated within the diverse Iranian society (Mazaheri et al., 2022). Commitment, loyalty, and trust are critical relationship quality and stability predictors. Couples who exhibit these qualities tend to have higher relationship satisfaction, intimacy, and resilience to challenges. Additionally, betrayal, whether through infidelity or other forms of disloyalty, can have devastating effects on relationships. Betrayal undermines trust, increases negative emotions, and can lead to the dissolution of the relationship if not addressed effectively (Baghaei and Yousefi Afrashteh, 2022).

Research posits that couples who engage in self-criticism or hold rigid beliefs about their partner's behavior may find themselves in a cycle of dissatisfaction and conflict (Adibkia et al., 2022). This aligns with Ellis's rational emotive behavior theory, which suggests that cognitive distortions contribute significantly to emotional distress within relationships (David et al., 2009).

Effective conflict resolution strategies can mediate the negative effects of irrational beliefs on marital satisfaction. By fostering open communication and understanding, couples can mitigate the impact of stressors arising from irrational thoughts. The ability to navigate conflicts constructively is essential for maintaining a healthy relationship (Tikdari Nejad and Khezri Moghadam, 2017). Our research revealed various strategies for resolving marital problems, such as reading books, engaging in honest conversations with your partner, seeking professional counseling, refraining from involving family members, cultivating patience, and remaining calm during conflicts. Recent studies align with the search results in emphasizing when needed to resolve marital issues. Tailoring interventions to the specific couple is also an important consideration. Couples should experiment with different methods to find what works best for them. Couples can effectively manage disagreements and enhance their bond by utilizing these proven techniques (Adibkia et al., 2022; Javdan et al., 2023).

### 4.3 Equity and upholding preexisting convictions

Our study identifies a category termed “Equity and upholding preexisting convictions,” derived from students' beliefs about marriage. This category encompasses three subcategories: embracing autonomy for women, male dominance, and religion, sexism, and cultural bigotries. Each subcategory reflects underlying societal attitudes and moral convictions that influence perceptions of equity within the marital context. Our research also demonstrated the beliefs related to women's autonomy in fostering equality within marriages. Conversely, beliefs in male dominance, as well as certain religious, sexist, and cultural biases, can harm relationships between spouses.

Women's autonomy, defined as their ability to make decisions and control their own lives, is a key factor in determining equality in marital relationships (Nepal et al., 2023). Research focusing on Chinese college students shows that, while there is a general interest in marriage, female students have lower marriage intentions than males, indicating a shift toward prioritizing personal choice and autonomy in marital commitments. This supports our finding that autonomy is gaining importance in students' beliefs about marriage (Xie and Hong, 2022). Additionally, another study emphasizes the role of women's autonomy in decision-making, particularly in financial matters, which empowers women and challenges traditional male dominance. Women who control economic resources experience enhanced self-esteem and negotiating power within households (Abbas and Rao, 2014). Recent scholarly investigations suggest that the spousal beliefs and attitudes, especially those of the husband, concerning gender roles and power dynamics within the marital union play a crucial role in influencing the degree of autonomy that women encounter in their marital partnerships (Sunmola et al., 2021; Zhao et al., 2022).

Historically, traditional households in Iran have played a significant role in shaping beliefs about women's autonomy. These households have been characterized by strong gender segregation, where household activities and social relationships are determined by gender, emphasizing concepts like confidentiality, purity, cooperation, and humility (Valibeigi et al., 2022). The traditional patriarchal extended family model in Iran, with the father as the absolute authority, has also influenced perceptions of women's autonomy within the family structure. However, recent societal changes, such as the modernization of social and economic conditions, urbanization, and access to education and social services, have started to impact the size, structure, and functions of traditional households in Iran, potentially leading to shifts in beliefs about women's autonomy within these (Salehi et al., 2020).

Moreover, our findings resonate with broader societal influences on marriage beliefs, particularly regarding religion and culture. A literature review on college students' views of marriage notes that cultural and religious factors significantly shape attitudes toward marriage and reproduction (Zhong, 2022). Furthermore, the impact of societal norms on perceptions of gender roles is evident in studies that explore how these norms can lead to sexism and cultural biases against women's autonomy in marital contexts (Musick et al., 2012). This supports our identification of religion and cultural bigotries as critical factors influencing students' beliefs about marriage.

Research found that Iranian culture strongly emphasizes family, with marriage being a culturally bound institution. Certain cultural beliefs and values, such as adherence to religious teachings, can influence marital quality and conflict (Sadeghi et al., 2012). Conversely, dysfunctional beliefs rooted in traditional gender norms lead to irrational expectations and confrontations between couples, negatively impacting their relationship. Rigid adherence to traditional gender roles, where women are expected to handle most domestic duties, can contribute to marital conflicts and dissatisfaction. Rigid beliefs about how one “should” behave can affect marital relationships. Shifting toward more egalitarian gender norms and sharing household responsibilities more equally seems important in promoting healthier marital relationships (Mohammadyari et al., 2023).

## 5 Limitations and strengths of the study

The main limitation of this study is that it collected data from a single university using only one data collection instrument at a specific period in time, which may have been influenced by the circumstances present at that moment. Additionally, selecting participants may introduce biases related to the sample size. These limitations can affect the study's generalizability and limit the applicability of the results to couples from different cultural backgrounds. We recommend that future research explore a wider range of participant contexts with a larger sample size. However, the strengths of this study are that it addressed all ethical issues, obtained IRB approval, and presented a strong discussion. The present study may open a new window for Iran to conduct more extensive research on students' beliefs about marriage.

## 6 Implications in psychosocial sciences

University students hold diverse beliefs on marriage, shaped by complex cultural, religious, and societal influences. Recognizing these key determinants is crucial for fostering healthy marital development among young adults. Educational interventions that raise awareness of these factors and integrate dialogues on intimacy, companionship, and equity into academic curricula can equip students with a more sophisticated understanding of marriage, facilitating informed decision-making and fostering positive attitudes. Educating students on establishing a secure marital atmosphere and providing strategies to tackle marital challenges can boost individuals' well-being and satisfaction in future marriages, aligning with evolving societal norms and expectations. Engaging in mutual thinking and discussions in the university environment can increase social interactions and exposure to successful married couples, positively influencing students' beliefs and perceptions about marriage. By addressing these key beliefs, higher education institutions can play a pivotal role in shaping their college population's marital beliefs and future outcomes.

## 7 Conclusion

Our research delved into the intricate network of beliefs influencing attitudes toward marriage among university students, highlighting the importance of “intimacy and companionship,” “establishing a secure environment,” and “equity and upholding preexisting convictions.” University student couples foster intimacy and companionship in marital relationships through beliefs related to empathetic connections and simplifying life, nurturing, keeping affection alive, and transparent communication. Cultivating a secure marital atmosphere in marriage is influenced by beliefs encompassing feelings of security and commitment, along with employing effective conflict resolution strategies. It is crucial for beliefs supporting women's autonomy to be present to enhance equality within marital partnerships. Conversely, beliefs in male dominance, some bigotries of religion, sexism, and culture can detrimentally impact spousal relationships. By examining these beliefs, our study offers a comprehensive insight into the diverse beliefs shaping marriage views, providing valuable implications for future research and practical applications in the psychosocial sciences. This study emphasizes the need for promoting positive and egalitarian beliefs to enhance marital quality and stability.

## Data Availability

The raw data supporting the conclusions of this article will be made available by the authors, without undue reservation.
