# Supplementary material for: Exploring marriage beliefs from the perspectives of married students
Source: Front Psychol. 2025 Feb 20;16:1481905. doi: 10.3389/fpsyg.2025.1481905 (PMC11882564; doi:10.3389/fpsyg.2025.1481905)
Supplement: Supplementary file 1 [file Table_1.docx]

**Supplementary Table S1: COREQ checklist**

| **Topic** | **Guide questions/description** | **Reported** | **Location in the manuscript (section, page no)** |
| --- | --- | --- | --- |
| **Domain 1: Research item and reflexivity** | | | |
| Personal Characteristics | | | |
| 1. Interviewer/facilitator | Which author/s conducted the interview or focus group? | M. Maddimeshat | Data collection  Page: 8 |
| 1. Credentials | What were the researcher’s credentials? E.g. PhD, MD | PhD | Title page |
| 1. Occupation | What was their occupation at the time of the study? | Researcher with a psychiatric and advanced nursing background and extensive work experience in the psychiatry department. She has experience in couples therapy and is certified in choice theory. | - |
| 1. Gender | Was the researcher male or female? | Female | - |
| 1. Experience and training | What experience or training did the researcher have? | During the interviews, the researcher held two qualitative research certificates and authored five articles on qualitative research methods. | - |
| Relationship with participants | | | |
| 1. Relationship established | Was a relationship established prior to study commencement? | Yes, The study details were explained to all participants. Taking part in the study was voluntary, and students could choose to leave the study without facing any consequences. | Method, Ethical considerations  Page: 10 |
| 1. Participant knowledge of the interviewer | What did the participants know about the researcher? e.g., personal goals, reasons for doing the research | All participants were informed of the study's purposes and stages, and their participation was completely voluntary.  In addition, informed consent was also signed by the participants for audio recording. | Method, Ethical considerations  Page: 10 |
| 1. Interviewer characteristics | What characteristics were reported about the interviewer/facilitator? e.g., Bias, assumptions,  reasons and interests in the research topic | The researcher is interested in the psychiatric field and has conducted two quantitative studies in this area and one qualitative study. She has experience in couples therapy and is certified in choice theory. |  |
| **Domain 2: study design** |  | | |
| **Theoretical framework** | | | |
| 1. Methodological orientation and Theory | What methodological orientation was stated to underpin the study? e.g., grounded theory,  discourse analysis, ethnography, phenomenology, content analysis | The present study is a qualitative study based on a conventional content analysis approach. | Method, Design  Study, page7 |
| **Participant selection** | | | |
| 1. Sampling | How were participants selected? e.g., purposive, convenience, consecutive, snowball | By purposive sampling method, 24 participants were recruited, including married medical students, who agreed to take part in the study. | Method, Participants, and Sampling, Page 7 |
| 1. Method of approach | How were participants approached? e.g., face-to-face, telephone, mail, email | Face-to-face | Method, Data collection, Page 8 |
| 1. Sample size | How many participants were in the study? | 24 participants | Method, Data collection, Page 8 |
| 1. Non-participation | How many people refused to participate or dropped out? Reasons? | After 21 interviews, data saturation was reached. Three more interviews were also conducted to ensure no other code or category emerged. None of the participants were refused or dropped from the study. | Method, Data collection, Page 8 |
| **Setting** | | | |
| 1. Setting of data collection | Where was the data collected? e.g., home, clinic, workplace | Married university students were interviewed in a comfortable setting in faculties. | Method, Data collection, Page 8 |
| 1. Presence of non-participants | Was anyone else present besides the participants and researchers? | No  The main researcher (M.M.) held one-on-one meetings in a quiet room with no one else present. | Method, Data collection, Page 8 |
| 1. Description of sample | What are the important characteristics of the sample? e.g., demographic data, date | Participants in this study were university students who volunteered and met the following criteria: 1. Male and female students interested in participating. 2. Must be married. 3. Must be currently studying. 4. Can be either undergraduate or postgraduate students. A total of 24 married university students (6 undergraduate and 18 postgraduate) from various faculties were selected through purposive sampling. | Method, Participants and Sampling, Page 8 |
| **Data collection** | | | |
| 1. Interview guide | Were questions, prompts, and guides provided by the authors? Was its pilot tested? | Married university students were interviewed in a comfortable setting in faculties. The main researcher (M.M.) held one-on-one meetings in a quiet room with no one else present. The research inquiry was developed based on existing literature(6, 7, 11), practical knowledge, and advice from academic and professional experts. We began the interviews with a general question: "What do you expect from your spouse?" Then we asked, "Do you think your expectations were reasonable?" Through active listening and a supportive atmosphere, the interviewer (M.M.) facilitated a safe space for the participants to express their narratives and viewpoints candidly. Two individuals' insights shared in pilot interviews who were not included in the main study, along with Lazarus's framework on marital myths (Lazarus, 1985), informed the direction of the subsequent interview questions (see Table 2).  During the interviews, the interviewer asked probing questions such as "Tell me more" and "Give me an example" to gather more information. Each interview lasted between 40 and 60 minutes, with an average duration of 50 minutes. | Method, Data collection  Page: 8 |
| 1. Repeat interviews | Were repeat interviews carried out? If yes, how many? | No |  |
| 1. Audio/visual recording | Did the research use audio or visual recording to collect the data? | Yes, Researchers collected data through audio recordings. | Method, Data collection  Page: 8 |
| 1. Field notes | Were field notes made during and/or after the interview or focus group? | We did not take any field notes during interviews because we did not use observation as our data collection method. Instead, we used different communication techniques for the interactive interviews and wrote memos and keywords during these sessions. | Method, Data collection  Page: 8 |
| 1. Duration | What was the duration of the interviews or focus group? | Each interview lasted between 40 and 60 minutes, with an average duration of 50 minutes. | Method, Data collection  Page: 8 |
| 1. Data saturation | Was data saturation discussed? | We conducted 24 in-depth interviews with married students and reached data saturation after 21, with the last three interviews yielding no new insights. | Method, Data collection  Page: 8 |
| 1. Transcripts returned | Were transcripts returned to participants for comment and/or correction? | In this study, the researchers’ long-term engagement, maximum diversity in sample selection, appropriate interaction with participants, integration of information, and checking of the data by the participants helped to increase the credibility. | Method, Ensuring Rigor, Page: 9 |
| **Domain 3: analysis and findings** | | | |
| 1. Number of data coders | How many data coders coded the data? | In this study,  confirmability was obtained by avoiding any bias and reaching an agreement on codes and themes by all research team members*.* | Method, Ensuring Rigor, Page:9 |
| 1. Description of the coding tree | Did the authors provide a description of the coding tree? | Before coding, we carefully read all the transcripts several times to understand the issues raised. We labeled each meaningful part of the text with a code. MM and SG then assigned codes to the condensed meaningful parts to more abstractly represent the participants' words. Finally, we grouped similar codes into subcategories and categories using a process of constant comparison, reflection, and interpretation. MM discussed all subcategories and categories with ES. In case of persistent disagreement, the judgment of a third researcher (SG) was decisive. | Method, Data collection, and analysis.  Page: 8-9 |
| 1. Derivation of themes | Were themes identified in advance or derived from the data? | Themes were derived from the data | Method, Data analysis.  Page: 9 |
| 1. Software | What software, if applicable, was used to manage the data? | The interviews were transcribed verbatim from Persian to English by MM and ES, and the data was transferred to MAXQDA 18 for better data management. | Method, Data analysis.  Page: 9 |
| 1. Participant checking | Did participants provide feedback on the findings? | Yes, checking the data by the participants helped to increase the credibility. | Method, Ensuring Rigor, Page: 9 |
| **Reporting** |  |  |  |
| 1. Quotations presented | Were participant quotations presented to illustrate the themes/findings? Was each quotation identified? e.g. participant number | Yes, category(themes) and subcategories were supported with direct quotes attributed to anonymized participants. | Findings |
| 1. Data and findings consistent | Was there consistency between the data presented and the findings? | Yes, the researcher made a good pairing between each quote with a code in the findings and highlighted them in parentheses. Also, the findings presented in the table help to clarify and ensure consistency of the data | Findings, text, and Table 4 |
| 1. Clarity of major themes | Were major themes clearly presented in the findings? | Yes, major themes (categories) presented Both in the text and in the table | Findings, text, and Table 4 |
| 1. Clarity of minor themes | Is there a description of diverse cases or a discussion of minor themes? | Yes, the researcher outlined the main findings and interpretations, highlighting both the similarities and differences compared to other studies. | Discussion |
